# The Ethics of Our Inquiry: An Interview with Hank Greely

**DOI:** 10.1371/journal.pgen.1004780

**Published:** 2014-11-06

**Authors:** Jane Gitschier

**Affiliations:** University of California San Francisco, San Francisco, United States of America

Sooner or later most scientists encounter a problem with ethical overtones. It could be as simple as what data to discard before submitting a paper or a question of authorship, but sometimes the quandary emerges from the very core of an experiment or in the application of newfound know-how to “advance” our society.

When I was a post-doctoral fellow, for example, I developed a DNA-based diagnostic test for the X-linked disorder classic hemophilia. The test meant that it would be possible to genetically predict hemophilia in the fetus of a pregnant woman who was known to be a carrier of the disease. Immediately I was asked to apply the test to a real-life situation: an Australian woman was pregnant, and DNA from the fetus, known to be male with a 50–50 chance of inheriting hemophilia, was sent to me. Those few days in the lab, now 30 years ago, of digesting the DNA and running a Southern blot were extremely stressful. I knew the very life of this child depended on my result; time was pressing and I struggled with my own queasiness over the appropriateness of what was being asked of me. Was hemophilia so devastating that an affected fetus should be aborted? On the other hand, abortion was legal and available with no reason needed other than the choice of the woman herself. I soldiered on, greatly relieved to predict that the baby would be unaffected and even happier to learn later that, indeed, a healthy baby was born.

My personal experience made me attuned to the many ethical issues that have arisen in the field of genetics in the years since then. Some current advances beg the question, “*Should* we do this, just because we *can*?” There are many aspects to consider in answering bioethical questions, including harm and benefit to individuals, born or unborn, their families, and society at large. For a broader discussion, I turned to Hank Greely (Image 1), a law professor who runs the Center for Law and the Biosciences at Stanford University. Greely previously worked on energy and gas law with an eye toward issues in global warming. He later became immersed in genetics questions when asked to serve on a panel discussion at Stanford and is now a member of the *PLOS Genetics* Editorial Board. We delved into his perspective after dinner, before a “live audience” at our recent Board meeting in San Francisco. With his engaging and gentle manner, his commentary softened the edges of some difficult issues and gave us a lot to chew on, starting with a disclaimer, after my introduction, that he isn't really a bioethicist.

**Figure:**
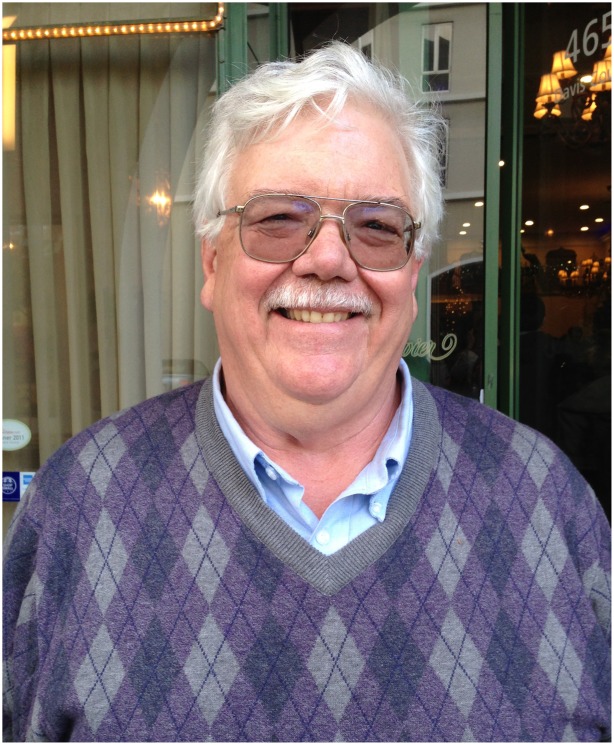
Hank Greely at the Editorial Board summit in 2014.

**Greely:** I don't think of myself as a bioethicist. I think of myself as a lawyer and law professor who works in bioethics.

There is a dispute about whether bioethics is a field (an area) or a discipline. An area—I think of something like Latin American Studies. You're an economist, you're a literature person, you're an anthropologist, you study Latin America. Whereas, philosophy is a discipline. Bioethics is an area with a bunch of interesting questions that people from different perspectives should approach. If I look at people who do what I think I do, they are people who have science backgrounds, medicine, anthropology, religious studies, law, sociology; I think there is even an economist. I view bioethics as a field of questions—moral, ethical, social, and legal questions—that come out of the incredible advances we are seeing in biology and their intersection with human society.

The reason that I push back against “bioethicist” is that ethics sounds like a cop or like a priest. You go to heaven, you go to hell, you go to purgatory and with enough *Ave Marias* we get you out early. Some of the people who define themselves as ethicists think like that, some don't. I think of myself differently. There are problems raised, tricky issues with pluses and minuses. Let's try to figure out a way of keeping society and the advances in biology in tune with each other.

**Gitschier:** I'd like you to just walk us through how someone who works on bioethical questions approaches them, because I suspect there is actually a logical system that is used. And I'll just pick an example. Let's say, and I know this isn't true, there were a single gene that conferred height and if you get a particular allele you'll be tall, and if you get a different allele you'll be short. You realize that in society there are some advantages for your children to be taller, but you and your partner are height-challenged. You want to give your future child an advantage by inserting the “tall allele” into his or her genome. How would an “ethicist” analyze this? Do you just knee-jerk respond, “Oh that's ridiculous?”

**Greely:** Of course! [Laughter]

**Gitschier:** Don't you have a thought process?

**Greely:** I can't tell you what an “ethicist” would do. There are a lot of ethicists and they have lots of different approaches, and I think that is one of the good things about the field. You can try to figure out whose approaches you like and respect and whose you don't. You should pay some attention to all of them.

Me? First, I try to figure out what is scientifically plausible and what isn't. Some of you may know about Ray Kurzweil and the singularity [from his book *The Singularity is Near*]. Some of Silicon Valley are in love with the singularity. That pretty soon nanotech and information tech and biotech will allow us all to upload our brains and personalities and live forever. I think it's total and complete bullshit. If it's not plausible science, I'm not interested in it. I want to try to understand what has a decent chance of working in the next 10, 20, 40 years.

Then I ask myself, if it did happen, what would be the consequences for society? And as complicated as biology is, law and society are even *more* complicated. We've got over 7 billion human beings on the planet, and each one wants to do exactly what he or she wants to do. All law, like biology, has exceptions and provisos with footnotes; everything is complicated.

My third stage is to say, which of these consequences will trouble people? Which will be things that people will say, “This is unethical, this is bad, this is evil, damaging, dangerous,” etc.? This is cheating a little, because I'm not saying, “Which ones do *I* find bad, evil, etc.?” I'm looking at what I think my *society* will find troubling.

And then given that, what kinds of interventions do we have to limit the troubling issues while allowing us to maximize the benefits? I used to be even more full of hubris than I am now, and I would say we want to maximize the benefits and minimize the harms. And over time I've come to think, if we could just avoid a few catastrophes that would be a good thing! Trying to figure out where are the landmines and how can we avoid disasters: that's what I think—and *hope*—I do.

**Gitschier:** Do you feel that you have some responsibility to come out and have some conclusion after all your ruminations?

**Greely:** Yes and no. I started as a lawyer. A lawyer is a servant; basically you are giving your client advice to help achieve the client's ends. You are the grease, the lubricant in the structure to help them see what they can and can't do. So part of me says my goal is to lay out the pluses and minuses, the benefits and the risks, and say, “OK, society, those are the pluses and minuses, the benefits and risks, you guys make the choice.” And if Germany wants to have a different regulatory regime than the United Kingdom, which wants to have a different regulatory regime than the People's Republic of China, part of me is OK with that.

Deeper than that, there are issues that I view as above my pay grade, and I just put on a shelf. Say, “free will.” I do neuroscience stuff too, and I cannot imagine how I can actually have free will, but it feels like I do. People smarter than I have thought about this for 2,500 years. So I'm just going to assume I have free will and not worry about it.

Then there is this deeper issue of fundamental human rights. It's a real problem, because if you think everything is culturally relative, if you don't care what China's regulations are or South Africa's under-apartheid regulations were, then maybe you could say, “Well maybe the Nazis were OK for their society,” and that can't be right!

On the other hand, if you say there are some inherent underlying human rights, you have to figure out what they are and you have to defend where they come from. And that gets hard, too, so I put that up there with free will.

There are some things I think are wrong—infanticide, let's say. I think infanticide is wrong. I can't prove that in any real way, but if one of the consequences of one of the things I was arguing about was infanticide, I'd say, “I think this is wrong.”

Things less than that, I'd say, here are the choices, you make the decision—you as a culture, as a government, as a society—decide what you want. Or you as individual parents, or as individuals, perhaps deciding whether to use enhancing drugs. I try to avoid the normative, but I can't entirely.

**Gitschier:** Have there been any things in the field of genetics that you feel are pretty black and white—“This is wrong, we shouldn't do this?”

**Greely:** Good question. In the field of human genetics, I think the things that worry me most actually revolve around safety. For example, about 15 years ago an in vitro fertilization [IVF] doctor in New Jersey did some mitochondrial transfer. I think mitochondrial transfer is an interesting technology and should be explored for a woman with a defective mitochondrial genome, which would be passed on to all her kids. She can't have healthy kids unless she uses a donor egg or unless she swaps out her mitochondria in her egg for somebody else's mitochondria. I think it is a potentially very interesting and valuable human suffering–reducing intervention.

But in 1999–2002 when the guy started doing it, he didn't even have any animal work to show this was a good idea. This was insanely risky and reckless. That's *wrong*.

I tend to be pro-science and relatively pro–individual choice. So the things I react very strongly against as being wrong tend to be safety risks, especially those not voluntarily chosen by the person put at risk. The baby who is going to be born from that egg that had the mitochondrial transfer never said, “Yeah, I volunteer! Transfer the mitochondria!”

It doesn't make me say we shouldn't do it. It makes me say let's make sure we have the preclinical studies as best we can and make sure we understand the mechanisms well before we start doing it.

**Gitschier:** Mitochondrial transfer has now been in the news again. Do you want to tell us a little bit about that, and this brings us to the FDA [Food and Drug Administration]?

**Greely:** Sure. It's an interesting history. The FDA in the United States is charged with regulating drugs, devices, biologicals, cosmetics, food additives, etc. And it has not regulated assisted reproduction to speak of.

**Gitschier:** In fact, that is an unregulated industry.

**Greely:** Almost entirely, in the US.

**Gitschier:** Which is shocking to me.

**Greely:** In other countries, it is substantially regulated. There are countries where you can't be a woman over a certain age, where you have to be heterosexual, a whole bunch of things that you can and can't do in almost every country. The US, all you need is a bank account big enough to pay for it.

**Gitschier:** Actually, I was referring to the technology itself. What's in the dish? My understanding is that assisted reproduction clinics don't have to report on what reagents they use, their protocols, etc.

**Greely:** The FDA has not regulated that.

Dolly [the cloned sheep] is now 17 years in the past, and you'll remember the uproar! Everyone jumped to the idea of “The Boys from Brazil” and cloning Hitler and armies of warrior slaves. There was great panic and politicians proposed a series of sweeping solutions to things that may not actually be problems.

The FDA announced that a cloned embryo would be a biological product, kind of like a vaccine, and would be subject to the FDA's regulation as a biological product. And so you couldn't try it without first getting an IND, an investigational new drug approval. And, by the way, the FDA said it is highly unlikely that they would grant such an IND. So that was really the FDA's first move toward regulating reproductive technology.

So, back to this guy in New Jersey with the mitochondrial transfer. After the FDA announced that a cloned embryo was a biological product that needed an IND, they said his embryos with the mitochondria transferred were biological products that required INDs. So he said the hell with it and he stopped it, after about 15 pregnancies! It's frustrating; we don't know what happened to those 15 pregnancies. The kids weren't research subjects and haven't been followed up very closely, if at all.

Fast-forward about 12 years: the OHSU [Oregon Health and Science University] group manages to do mitochondrial transfer in a monkey species—successfully—and they tell the FDA that they would like to start doing clinical trials in humans. Now the US tends to think it is the only place in the world, but the UK had been considering this several years earlier. The Nuffield Council, which does bioethics reports there, had considered it and said, “This could make sense.” I believe they are planning a vote in Parliament, a free vote, a conscience vote, on whether to amend the statute creating the Human Fertilisation and Embryology Authority to allow this kind of thing. And my understanding is that there is a good chance it will pass.

In the US, these guys from Oregon went to the FDA and said, “We'd like to try this,” and so the FDA told its advisory committee on reproductive technologies to look at it. They held a two-day hearing in February, and as far as I can tell much of the hearing was quite substantive. What are the risks? How much animal work have you done? What could you learn from human embryos in vitro?

But all the press was on the ethical concerns, like any baby born this way would have—wait for it—*three* parents. Or, “This might introduce new mutations into the human germline.” There were a bunch of silly arguments that got taken seriously.

If you talk to a philosopher, “ethics” means something very specific, what philosophers and only philosophers do. Ethics in other contexts means genuine moral and ethical dilemmas, public relations, politics, a bunch of stuff, and a lot of the stuff that gets played as ethics, like the idea of “three parents,” if you push the logic very far—we actually have DNA from four people, and eight people and 16 people, and so on—there's not a lot there. I love it when people flying across the country in airplanes, sitting in a closed room with artificial lighting, or using PowerPoint, inveigh against “unnatural” stuff. This is not how our ancestors lived 80,000 years ago! *Everything* is unnatural.

I think that a lot of things that get called “ethical” questions are visceral reactions that become politically charged. And I think part of what good bioethicists—people who work in bioethics whom I respect—do is to try to sift out the genuinely tough questions from the gut-reaction, “yuck-factor” questions.

**Gitschier:** In the 20 years or so you have concerned yourself with biology, what do you think have been the genuinely toughest ethical questions?

**Greely:** I'll give you two: one continuing from the past and the second one—a big one—I see starting now and continuing into the future.

The thing in the past has been research subjects and the extent or lack of extent of their control over the DNA samples they give people. It's a deep ethical problem, because there are two good principles at war. Relieving human suffering is a good thing, and I do believe that biomedical research will relieve more human suffering than it will cause. But for people to know what is happening with their family histories, with their medical records, with their DNA is also a good thing. And the problem is that, especially as you go for bigger and bigger numbers [of subjects], getting people to give anything near to real informed consent is time-consuming and expensive. It also becomes a confounding variable in terms of who agrees and who doesn't. The perfect thing would be to take everybody in the country and look at all of their health records and mine all that data. And yet a lot of those people are going to disagree.

For example, about 20 years ago a professor from Arizona State went to the Havasupai, a native American tribe from the Grand Canyon region, who have a problem with diabetes, to look at their DNA. Twelve years later, one of the tribe was at the University and was invited to listen to the oral defense of someone who had used some of these data. The visitor was appalled to discover the data had not been used for research just about diabetes, but also about their ancestral origins in Asia. The Havasupai *knew* their ancestors had always been in that canyon, that they were created there, yet their DNA had been taken and used to support something that was completely different from their cultural story.

Another recent example: In the US we take blood spots of newborns to test for early onset diseases, and some states save them, some don't. These blood spots are taken not only without parental *informed* consent, but without their consent at all. It is illegal *not* to take a blood spot in California. Texas had saved about 5 million blood spots, and they were used in research by the FBI for forensics, just to get population estimates for CODIS [combined DNA index system] markers. But the parents were outraged when they learned of this. It resulted in a lawsuit, and Texas passed a statute saying that any research [involving the spots] has to be done with parents' informed consent. And that the 5 million existing blood spots had to be incinerated. Up in smoke.

One last example: Group Health Cooperative of Puget Sound and the University of Washington [UW] were doing a study of Alzheimer disease, looking at first-degree relatives of people with Alzheimer and getting their genotypes. At some point the NIH said, “We're funding this, we want you to put these genotypes into dbGaP” [database of Genotypes and Phenotypes], the world's ugliest acronym. UW didn't complain, but Group Health did. They said, “Wait, our members might be upset by this; it didn't say this in the consent form, and we can't deposit it unless we consent.” NIH agreed to pay for re-consent, and it also agreed to pay for some bioethicists to study the re-consent process. The researchers found 1,400 of the original subjects who were still “cognitively intact” and they asked them to consent for the genotype banking. The good news for science is that 88% agreed to let their data go into dbGaP, but that means 12%—not a trivial percentage—did not agree. The researchers also interviewed a subset of people who agreed to let the data go in; 90% said it was important to them to have been asked.

What I'm worried about [for large genetic studies] is a Texas kind of reaction. There are now nearly a million people in dbGaP, many of whom don't know they are in dbGaP or know what that means. And you can say we can't re-identify them, but we know we can get not only their whole SNP array but also a lot of phenotypic information. Fundamentally, the more useful the information is, the more identifiable somebody is.

I do feel that in the long run, there *is* no privacy and we need to get over it. But right now people really care about this, maybe more than they should. I'd much rather let you see my genome sequence and my health records than my credit card billings or my Google searches. People think their health information is much more private and sacred than it is. They care about it, and a mismatch between public understanding and expectation, on the one hand, and reality, on the other, is potentially a catastrophe, as I think it was for the blood spots in Texas. So I think this is the biggest past and current issue.

**Gitschier:** And the upcoming issue?

**Greely:** It gets back to your initial question about height. Parental selection of children based on their genetic traits. Prenatal diagnosis has been going on for 40 years, but it has been limited largely to chromosome abnormalities, a specific Mendelian disease, or sex determination. Now that we can do whole genome sequencing on a single cell, you can see *everything* about your unborn.

Also, it used to be that you had to have an invasive procedure—amniocentesis or chorionic villus sampling—unpleasant, expensive, with a small chance of pregnancy loss, and in the US, only about 1%–2% of the babies are tested in this way.

However, non-invasive prenatal testing [NIPT] is now a possibility. If you are a pregnant woman, by about the eighth week of pregnancy, 5%–10% of the cell-free DNA in your blood is not from you, but from the fetus. There are now four companies in the US and several overseas that will give you a pretty good answer about aneuploidies, just from your blood. A couple of companies are doing sex-chromosome aneuploidies, which is a little more worrisome because how serious is Turner (XO)? How serious is Klinefelter (XXY)?

This was first done clinically in October, two-and-a-half years ago. Last year, 500,000 women around the world got this test! This year there were probably 500,000 in the US alone. In the long run, we're looking at 50%-plus pregnancies in the developed world getting this information.

There is proof of principle now [with NIPT] for single-gene diseases. Both individual women and couples, on the one hand, and societies, on the other, are going to have to make decisions about what they want to do.

And I don't think NIPT will be the end of it. In 20–40 years most babies in the developed world will be conceived in IVF labs so parents can do genetic diagnosis by whole genome sequencing of pre-implanted embryos. They'll make a hundred embryos, and decide which one or two to transfer. A *hundred* embryos, how are you going to do that? I think we'll be able to take skin cells, turn them into induced pluripotent stem cells [iPSC], which we've done already, differentiate them into embryonic stem cells, which has been done in mice, mature those oocytes in vitro, which we have been able to do in humans for quite a while, and then you have an unlimited supply of eggs without egg harvest and the expense of IVF, which is the cost of the hormones and egg retrieval and the risks.

**Gitschier:** You are also doing away with an age limit.

**Greely:** That is right—in both directions! An 80-year-old can produce fertile eggs, so can a three-year-old. The question is whether we will be able to turn iPSCs safely and effectively into mature oocytes.

**Gitschier:** It's just a matter of time.

**Greely:** I'm actually writing a book on this topic with a kind-of catchy title, “The End of Sex”, but what disturbs me is that my wife really likes the title!

This technology will present a lot of challenges, on two different levels, personal and societal: Would you want to do it, and would you want it regulated or banned? How far are we willing to go or to let others go to make babies, in choosing their genetic traits even if you don't want to?

Now this audience understands that genes, although they are important, are not all *that* important. Monozygotic twins are not the same people. Parents who expect their kid to be a great quarterback based on the genome they pick are likely to be disappointed, ‘cause there is a lot of other stuff that goes into it. But parents will want to do it; how far we will let them do it and what will be the political pressures to restrict it are going to be the big genetics issues from now to the next 30 years or so.

Several American states have banned abortion based on sex or race. That has not yet been challenged in court. And it's unclear how enforceable that is. North Dakota just passed a ban on abortion based on disability that's been enjoined by a federal court. In North Dakota, a woman is not allowed to abort a Down syndrome fetus. This is going to be a hot issue in a lot of countries.

And with CRISPR technology [i.e., the ability to insert new genetic material into our genome via a technology based on “clustered regularly interspaced short palindromic repeats”], you can imagine parents not being limited to their own alleles.

**Gitschier:** Right. That was my point about the adding the theoretical “tall gene” into genomes of embryos from short parents.

**Greely:** I personally have a range of reactions to that. There are allelic-interactions risk factors involved, and then risks from the process used to modify the genome. I think the riskiness of genetic engineering at the embryo stage is sufficient that I view that as several decades off from the selection [of unaltered embryos]. I'm not going to live to see it. By the way, piece of advice, if you're writing a book with a big prediction, time it…

**Gitschier:** …so you're dead.

**Greely:** Time the prediction for 40 years so you're unlikely to be proven wrong.

I think the decision is really interesting, and really hard, but is not my decision to make; it is my grandchildren's, and their views of the world and the universe and ethics may well be different from mine. And in their world and in their time, their views should rule and not mine. And in a way this gets back to the deeper issue of cultural relativism versus universal human rights. Except at the extreme, I don't think we should accept that what we think is right is at some deep core level right and that future generations won't think differently.

I look at my own lifetime and look at the changes that have happened in my country in terms of African Americans, women, and the one that blows me away the most, gays and lesbians. Within the blink of an eye, the culture has changed dramatically. Years from now if genetic modification becomes possible, will people hate it, will they love it? I don't know. But that is one of the things that make these such interesting questions: they don't have permanent answers.

